# The Hospital. Nursing Mirror

**Published:** 1898-09-03

**Authors:** 


"The Hospital" Nursing Mirror.

Being the Nursing Section of "The Hospital."

[Contributions for this Section of "The Hospital" should be addressed to the Editor, The Hospital, 28 & 29, Southampton Street, Strand, London, W.C., and should have the word "Nursing" plainly written in left-hand top corner of the envelope.]

### News from the Nursing World.

### THE PRINCESS OF WALES AND THE INNISKEA NURSES.

On Saturday last the Lord Lieutenant of Ireland and the Countess Cadogan gave a garden party on the occasion of the admission as "Honorary Serving Sisters" in the Order of St. John of Jerusalem of the nurses of the City of London Nursing Institution who distinguished themselves in nursing the typhoid-stricken at Inniskea last year. The nurses owe this honour to the interest taken in their work by H.R.H. the Princess of Wales. The Countess of Arran, wife of the Lord Lieutenant for County Mayo, personally presented the Princess with a memorial largely signed by influential persons, and she suggested their admission to the Order to the Prince, who is the Grand Prior.

### THE RED CROSS IN EGYPT.

Colonel Young, Commissioner of the "British National Society for the Aid of the Sick and Wounded in War"—which is the full title of the British Red Cross Society—recently left for Cairo, and is busy organising supplementary help for the sick and wounded in the present campaign. On Friday last a telegram was received at the London head-quarters asking for three trained nurses, and on the 29th inst. three members of the Nurses' Co-operation set out. They are old and valued nurses on the staff of the Co-operation, and their names are Miss Isabella Gibson, Miss Elizabeth Geddes (both trained at the Dundee Royal Infirmary), and Miss Anna Burke, who was trained at the Royal Berkshire Hospital. It is expected that they will arrive at Alexandria on Sunday next, where they will be met by the Commissioner or his substitute, who will give them instructions as to their destination. Their work will be to bring the sick from Assouan by steamer to Cairo, where they are to be met by the regular army medical staff, and nursed by the Army Nursing Sisters in the Citadel Hospital. The journey for invalids from Khartoum to Assouan will probably occupy ten days, and from Assouan to Cairo by steamer another ten days. The present campaign has been very carefully thought out, and the military medical ordinance is replete with all the necessities that will be required, so that the part taken by the society will be rather to supplement than to take an active share in the work on hand. The British Red Cross Society has no other operations on foot just now, nor did it take any great part at the time of the hostilities between Greece and Turkey, as there were many agencies covering the same ground. The authorities contented themselves, therefore, in administering relief through the Ambassadors principally to refugees.

### NURSING AT ST. GEORGE'S.

The nurses' accommodation at St. George's Hospital is in the process of rearrangement. The cubicles which have hitherto served as sleeping apartments are gradually being abolished and separate bedrooms substituted. It is a work of time; as the British workman is in possession, and it can only be taken in hand bit by bit. The hospital possesses a nurses' home in Montpellier Street where most of the senior nurses live when off duty; there the matron has at her disposal a few bedrooms, which she is using whilst the alterations are being made in the hospital. As one batch of nurses takes up its abode in its new quarters in the main building another goes into residence at the home. Some little time ago it was reported that the nursing staff was dissatisfied with the appointment of sisters who had not been trained in the hospital. The matron informs us that no permanent appointments have as yet been made. What has happened is that, five sisters have left—one for South Africa, one to the Fever Hospital, Banstead, and others for private work—and their places have been filled temporarily by outside nurses, because it was impossible to adjust affairs at the moment, and none of the permanent staff could be spared to take up the work. The matron is on the point of taking her holiday, and when she returns and things are settled more definite information will be available. Nurses Plaxton and Leitch, both trained at the hospital, have accepted appointments at the Municipal Hospital of Rangoon. They are the first English nurses employed by the hospital, and their work will be to undertake the care of plague cases if plague breaks out, and if happily it does not to take part in the general nursing of the hospital.

### RESIDENTIAL HOME FOR THE NURSES OF THE CO-OPERATION.

Ever since the Nurses' Co-operation has become such an undoubted success, the want of a residential home for the nurses has been much felt. Thanks to careful management, a substantial sum stands to the credit of the members of the Co-operation, and a desire has always existed to invest this sum in a manner to secure greater comfort to the nurses employed by the Co-operation. No one was more desirous of securing a home for her nurses than the first lady superintendent, Miss Phillipa Hicks. She was much disappointed when an excellent opportunity to acquire a valuable property was not taken advantage of by the nurses. Her excellent work on behalf of the Co-operation was recognised by Lady Howard de Walden, who generously offered her assistance to Miss Hicks to carry out her much wished-for scheme. The desirability of securing a site as near the Co-operation as possible has caused some delay, and, in the meantime, Miss Hicks has had to abandon the scene of her labours. Her interest, however, she retains, and she will be closely associated with the new institution. The home, which will be situated in Langham Street, will probably contain accommodation for 30 nurses simultaneously—accommodation regarded as sufficient for the staff of about 400, who are very constantly employed. Lady Howard de Walden's generosity will take the form partly of a gift, and partly of a loan, to supplement the savings of the nurses. The scheme will probably be vested in trustees, and a separate committee of management will be appointed.

### PLAGUE ONCE MORE IN BOMBAY.

As was feared with the change of weather, there has been a further outbreak of plague in the city and presidency of Bombay. Unfortunately, of the three Europeans that have been attacked lately, one (Dr. Elizabeth Philips) is dead.

### TRAINED VERSUS UNTRAINED NURSES.

Last week the fate of the Sisters of St. Dominic, who had to return home to be duly trained, after having nursed for seven years through the campaigns of Rhodesia, was chronicled; this week that of the matron of the Cooma Hospital, New South Wales, comes to notice. The hospitals in Australasia are mostly subsidised or maintained by the State, and that of Cooma receives £79 half-yearly from that source. As it happens the matron, who has been there for the last six years, is not certified; and the chief medical officer of the Board of Health has permitted the usual half-yearly subsidy to be paid only on condition that the Hospital Committee shall engage a fully-trained nurse for the position before the next payment becomes due. At present the committee decline to dismiss her, and it remains to be seen if they can afford to sacrifice £158 a year in order to retain an officer who does not fulfil the requirements of the law as it stands after the passing of the Hospital Act in 1894. That those in charge of the nursing of any establishment should be thoroughly qualified for their work is sound common sense, and in every way merits approval. Unfortunately, progress in any direction frequently presses hardly on individuals, and it is by no means an easy task for the authorities to be just to the institution over which they rule, and at the same time kind to the individual who must be displaced. Perhaps the matron of the hospital at Cooma will follow the example of the Irish nuns and obtain leave of absence whilst she acquires the necessary certificate. It is quite possible for her duties as matron to be discharged by a substitute for the time being.

### NURSING IN BELFAST.

The scheme of the Belfast Board of Guardians with regard to training nurses for the workhouse hospital has been partially accepted by the Local Government Board for Ireland. It may be remembered that the Guardians proposed to appoint probationers at certain salaries for three years, and at the end of that period to promote them to be nurses. The Local Government Board, however, direct the attention of the Guardians to the fact that they have not made any provision for the due instruction and examination of the nurses. At the debate at a recent meeting of the Guardians the chairman asked "if there were any examining body in Belfast"? and Mr. Agnew, the inspector of the Local Government Board, inquired "whether some arrangements could not be come to with the Queen's College authorities to examine the nurses," as there is no examining body for nurses in the three kingdoms. Mr. Oswald proposed a resolution recommending the Local Government Board to appoint as an honorary examining board the three visiting medical officers, with Dr. Campbell, Dr. Stafford Smith, jun., and Dr. Brice Smyth, jun. It was also decided to advertise for fifteen probationer nurses. In the end, the Guardians will have a recognised training school for nurses at the Belfast Union Hospital; and, having been obliged to take this step, they would only be acting kindly as well as wisely by all parties concerned—public, patients, and probationers—if they took pains to make it a first-rate one.

### HEBDEN BRIDGE NURSES' HOME.

About £1,546 were raised at Hebden Bridge as a memorial of the Diamond Jubilee. The greater portion of this sum has been expended in the purchase of two houses in Croft Terrace as a nurses' home. The Co-operative Society kindly gave suitable furniture, and on the 18th inst. the committee had the pleasure of formally installing the two nurses now at work in the district in their new abode.

### HUMOURS OF BUMBLEDOM.

The Guardians of Loughrea, Dublin, and the medical officer are at loggerheads, and this is the reason: The medical officer represented to the Guardians that the appointment of a trained nurse was advisable, and they disagreed with him. In the course of events a critical operative case occurred, and the doctor secured a qualified nurse to attend it, who, because of the lack of accommodation in the house, was compelled to lodge at the hotel. In the end the Guardians were presented with a bill of £5, in addition to the nurse's fee, for her board and residence. They not only declined to pay it, but ordered the doctor to do so himself, as they were not consulted as to the nurse's engagement, and by fourteen votes to two censured the doctor for his action. The Local Government Board has intervened, and, approving the doctor's action, declares that the responsibility of making such appointments rests with the medical officer and not with the Guardians, and that consequently the vote of censure was not deserved, nor is he responsible for the outlay involved. Perhaps the Guardians will surrender at discretion, and avoid similar causes of friction by appointing a qualified nurse.

### NEW NURSING ARRANGEMENTS AT ASTON AND HULL.

The Aston Board of Guardians held a meeting at their workhouse, Gravelly Hill, on the 9th inst., and discussed a letter from the Local Government Board commenting on the inadequacy of the nursing staff for the requirements of the workhouse. It appears that there is accommodation for 582 patients in the sick wards, and the number of inmates at present is 400. The nursing staff consists of a superintendent and 17 nurses, which gives each nurse, including the superintendent, 27 patients to take care of. If the wards became full a nurse would have to look after 39. As a matter of fact each day nurse does attend to 39 sick. It was resolved to appoint eight more nurses at the usual salary of £30 a year, and that probationer nurses should be trained for three years, at salaries of £10 for the first, £14 for the second, and £18 for the third year. The difficulty experienced by the Hull Guardians in obtaining charge nurses has led them to consider the advisability of appointing a resident medical officer and superintendent nurse, by whom the probationers could be trained. The chairman of the Hospital Committee explained that they had been somewhat unfortunate in their superintendent nurses, but now they had one who, with the medical officer, was prepared to give instruction to the probationers, which statement was well received.

### BENEFACTION TO THE NURSING SISTERS OF ST. JOHN THE DIVINE.

An anonymous donor has given the nursing sisters of St. John the Divine the sum of £5,000.

### Antiseptics for Nurses.

By a Medical Woman.

### XVI.—THE CAMPHORS.—ESSENTIAL OILS.—BISMUTH AND ITS COMPOUNDS.

The camphors belong to the group of oxidised essential oils. Ordinary or laurel camphor is obtained from the laurel camphor tree by boiling the wood in water, and condensing the camphor as it passes over and finally purifying it by sublimation. An oil distils over during this process, called oil of camphor or liquid camphor, which, by exposure to the air, oxidises and deposits common camphor. This occurs as the well-known white crystalline solid substance, which volatilises readily at ordinary temperatures, and combines readily with bromine and iodine, but not with chlorine. Camphor is only very slightly soluble in water, since only about 1 per cent. dissolves in it, thus forming the liquid that is popularly known as camphor water, and to which the camphor has communicated both its own special odour and its stimulant and antiseptic properties. It is, however, very soluble in alcohol, and in this form it acts as an irritant to the skin, and also forms fluid compounds with carbolic acid and thymol. When ignited, it burns with a luminous, sooty flame, and when burnt alone, or with some spirits of wine, the products of combustion contain little if any camphor. Spirit lamps containing camphor and essential oils in spirit have often been proposed for the fumigation of rooms after infectious diseases, but they would obviously be of very little use, since even by this means there cannot be produced sufficient of the vapours to disinfect the air properly, and, moreover, even when pure, camphor cannot be regarded as a powerful disinfectant. It might be very useful as a deodorant. There are various preparations in which camphor occurs as an ingredient, of which we give the following:—

Sanoscent.—This is a black disinfectant substance, and is composed of camphor, eucalyptus, pine oil, and other similar aromatic oils. It is used as the basis of most of those so-called disinfectant blocks and cakes commonly met with, and which also contain either naphthalene or paraffin and plaster, &c. Reference has already been made to the blocks when treating of the naphthols.

Hebden's camphor tar.—This contains camphor, eucalyptus, and tar distillates. It likewise is prepared in the form of blocks and cakes for use in lavatories, &c., and is also recommended to be used grated into a powder and sprinkled amongst clothes for protection from insects, &c.

A mixture of equal parts of camphor and charcoal has also been recommended as a disinfectant. Also camphor and menthol, camphor and magnesium, camphor and calcium chloride, have been recommended by different authorities at various times, for disinfecting drains, but they have never been widely adopted. A combination of bromine and iodine with camphor forms a very powerful antiseptic, but it is extremely irritating to the throat and eyes, which forms an insuperable drawback to its use, consequently, with so many other equally efficient disinfectants in the field which are at the same time free from this particular drawback, bromo and iodo camphor is merely a curiosity and not generally used. Moreover, when it is combined with such a strong disinfectant as either bromine or iodine, camphor itself would seem to be an unnecessary addition, as it can hardly increase perceptibly the disinfecting properties of its more powerful ally, and hence is superfluous. Camphylene, a disinfectant which has been recently introduced, can be obtained as either a liquid, solid, or in powder; it is said that it undergoes continuous evaporation at ordinary temperature when exposed to the air, sunlight, &c., and that the vapour so given off will destroy micro-organisms in the air; also that its solid form is non-poisonous and non-corrosive. The accounts of the properties of any new disinfectant, as set forth by the inventor, must, of course, always be accepted with caution, but camphylene can be recommended as a fairly good antiseptic and as an excellent deodorant.

Many other essential oils also possess some antiseptic properties, but the expense involved in their production will prevent their being generally used, especially in these days of cheap and effective disinfectants in great variety. Amongst the more important and better known of these essential oils we may mention the following: Menthol or oil of peppermint contains a hydro-carbon called menthene, which possesses distinctly antiseptic properties. It is a useful antiseptic, and is said by some authorities to be superior to phenol. It is sparingly soluble in water, but readily so in alcohol.

Oil of cloves is heavier than most of the essential oils, and rather more soluble in water and more volatile, all of which properties increase its value as an antiseptic. Oil of cloves consists chiefly of a liquid terpine, holding in solution eugenic oil, which chemically resembles phenol, and a variety of camphor, which is known by the name of caryophyllin, as well as a salicylic compound.

Oil of caraway is another heavy essential oil. It contains a hydro-carbon called carnol, which resembles thymol chemically, and which when dissolved in an alkaline solution of ammonium sulphide, deposits a peculiar substance called carnol hydrosulphide in yellow crystals, which is insoluble in water and sparingly so in alcohol, and has a very disagreeable smell. It is, however, strongly antiseptic, though not available on account of the cost and trouble of its production, its insolubility, and its poisonous characteristics.

Bismuth in itself has no special disinfectant properties though it is said to prevent fermentative changes, and hence one of its uses as an internal antiseptic. There are, however, three useful disinfectants which contain bismuth, called thioform, aerol, and xeroform, of each of which we propose to give now a short account.

Thioform is an absolutely non-poisonous substance which can be produced more cheaply than iodoform, and is said to give results far superior to those yielded by the latter, or even by carbolic, but this is hardly possibly, as it does not contain any powerful antiseptic, and can only owe such properties to the presence of salicylic acid. Thioform is a salt of bismuth, containing in addition sodium and salicylic acid. It is a yellowish grey powder, quite inodorous and tasteless and very insoluble in water, alcohol, and ether. The way in which it acts is to rapidly dry the wound and cover it with an impermeable layer, and so prevent the entrance of pathogenic organisms from without. Hence, it can only be regarded as a protection, similar to plaster.

Aerol has for its basis gallate of bismuth with some iodine combined. It is a greyish-green powder, entirely odourless and tasteless, and does not decompose on exposure to light. It has been used with success on the Continent, and has given satisfactory results, especially in wounds accompanied by much secretion. It does not irritate the skin.

Xeroform is a combination of bromine, phenol, and bismuth, and is intended to replace iodoform, of which it has none of the disadvantages, and is said to be as good an antiseptic. Professor Heuss speaks very favourably of it as a non-toxic, non-irritating, and, hence, useful application for the skin and mucous membrane, and absolutely without either taste or odour. It combines the properties of phenol and bismuth—to the former it owes its bactericidal and anti-putrid action, and to the latter its power of controlling secretions when excessive. It is said to be specially valuable as an intestinal antiseptic, and also as a protective application for wounds, of which it favours the cicatrisation.

### The British National Red Cross Society.

VERY little appears to be known of the British National Red Cross Society, and a few particulars of its work will, therefore, not be unacceptable. In the first place its correct title is the "National Society for Aid of the Sick and Wounded in War," and the last report was published at the close of the Egyptian campaign of 1884-5. Since the Russo-Turkish war, when the society expended rather more than £30,000, help has been given to the sick during the Zulu war; in the Soudan; and twelve nurses have been trained at Netley under its auspices, who have been drafted into the regular service under the new regulations for army nurses which came into force on April 1st, 1885. In March, 1879, a meeting of the executive committee was held for the purpose of considering what help could be rendered to the soldiers serving in the Zulu campaign, and a commissioner (Deputy-Commissary J. S. Young) was credited at Natal with a sum of £1,727 for the "purchase of medical comforts, hospital equipment, clothing, and newspapers for the recreation of the soldiers in hospital, and in grants to the extent of nearly £400 to local funds, to medical officers in charge of homeward bound transports, and to nursing staffs."

Major Young also acted for the society during the Boer Rebellion in 1881, when £1,100 was spent in alleviating the sufferings of the soldiers. It was during this campaign that the value of trained nurses became so apparent, and the society undertook the instruction, at Netley, of the twelve ladies who are now in the army service. In October, 1884, the committee once more appointed Assistant-Commissary-General J. S. Young as its commissioner for the operations in Egypt, with instructions to render all possible supplemental aid to the army medical officers. He was joined at subsequent dates by Dr. E. F. White, Mr. J. Dale, and Dr. W. H. J. Brown. The society, after some initial difficulty, succeeded in obtaining a launch drawing only two and a half feet of water and of steaming five miles an hour against the stream. She was christened by Lady Baring the "Queen Victoria," and another, which was bought later, was called the "Alexandria."

When military operations were extended to Suakin the society established a branch there in the care of Mr. V. B. Kennett-Barrington, and Sir Allen Young, C.B., generously placed his yacht, the "Stella," at his disposal for the purpose of taking the sick and wounded from that place, and also giving suitable patients the benefit of short sea trips.

Later four nursing sisters joined the branches, Sisters Hicks and Dowse being attached to that in the Nile district under Major Young; and Sisters Wrigley and Machen to that at Suakin under Mr. Kennett-Barrington. These sisters were in the pay of the Princess of Wales's Branch, which is affiliated with the National Aid Society.

All connected with the society fulfilled their mission with great credit, and were cordially welcomed and assisted by the civil and military authorities with whom they came in contact.

An interesting history attaches to the second of the launches—the "Alexandria." At the conclusion of the campaign the society presented her to the Government, and one morning, at three o'clock, she sank. Fortunately, there was no loss of life, as the invalids and staffs on board succeeded in reaching the banks of the Nile, off which she was lying. The "Alexandria" was raised, and is now in use. In a few days she will probably be of the greatest value to the poor men who will be wounded in the battle that is imminent.

Colonel Young is once more in charge of the society's affairs at Cairo, and three highly-trained and competent nurses are on their way to serve under him.

There is much satisfaction in feeling that the National Aid Society is on the alert in case of need, but there is almost more in knowing that the army medical organisation is so nearly perfect that it only needs to supply extra comforts and not necessities. This is as it should be.

### The Plaistow Nurses Again.

THE *Lancet*, August 27th, has a strong article upon the "Maternity Charity and District Nurses' Home" at Plaistow, dealing more especially with the intrusion of the Plaistow midwives into the practices of the medical men of the district. Incidentally, however, it lays bare an evil in regard to nurses which is undoubtedly of a very flagrant character. Far better that both midwives and nurses should be trained in properly equipped hospitals than in outside maternity charities, if training in the latter, which, according to the description given by the *Lancet*, seem to be almost commercial concerns, involves such proceedings as are here described. Writing of the Plaistow nurses, the *Lancet* says:—

"Their business should be to nurse the poor gratuitously, but to nurse the poor alone. If they nurse persons who can afford to pay then they are unfairly competing against professional nurses who depend for their livelihood on the fees which well-to-do persons can give. The position of a well-trained nurse is as worthy of consideration as that of any other member of the community, and it is gross abuse that money given for charitable purposes should be employed to ruin so estimable and useful a calling as that of a trained nurse. Yet many high-class, or at least wealthy, people in the eastern suburbs of London employ nurses from the Plaistow Maternity Charity for various kinds of illness. For this ordinary nursing the nurses are not allowed to take any payment, but when the people who employ them have means they generally make a donation to the charity. Such donations are payments in disguise. From every point of view this is a heinous system. A person is ill; he has means, and he wants a nurse. The only honourable course to pursue is to employ a trained nurse and pay the full market value for her services. Instead of this the patient sends to a charity, takes a nurse away who ought to be attending to the poor, thus defrauding the poor of the help which this nurse could give them, and for which charitable persons have subscribed their money. The rich man, after having been nursed gratuitously by the charity nurse, now steps forward as a philanthropist by making a donation to the charity, giving probably a sum inferior to that which an independent trained nurse would have cost him. Far from being a philanthropist, this man has been favouring a system of undercutting the market for trained nursing, thus helping to ruin the trained nurses. On the other hand, he has withdrawn from the slums of London one of the nurses available for the nursing of the extreme poor. He has saved a few shillings at the cost of the poor and at the cost of the trained nurses; and yet he appears on the list of donors to a charity, and those who are not acquainted with the inner workings of the transaction would look upon him as a philanthropist. This has happened over and over again, and it is quite possible that many who have committed this abomination are in no wise conscious of having done anything wrong. In this commercial community the instinct to buy in the cheapest market is so strong that it is done automatically and without reflection as to the moral consequences and responsibilities. But while it is thus scarcely surprising that some members of the general public should with thoughtless ignorance act in this manner it is most astonishing that a charitable and nursing institution should lend itself to the perpetration of such gross abuse. Here is the Plaistow Maternity Charity taking fees of from £15 to £40 to train poor girls as nurses, and yet it employs these very nurses to undercut the market and render the profession of nursing more and more precarious and ill-paid."

### Investiture of the Inniskea Nurses.

On Saturday last an interesting ceremony took place at the Viceregal Lodge, Dublin, on the occasion of the eleven nurses who distinguished themselves in nursing the epidemic of typhus on the island of Inniskea during June, 1897, being invested with the Order of the Hospital of St. John of Jerusalem in England, in which Order they have been accorded the rank of "Honorary Serving Sisters." The badge worn by members of this Order is an eight-pointed Maltese cross, ornamented with alternate lions and unicorns at each of the principal angles. A number of noteworthy persons assembled to take part in the function—hitherto unknown in Ireland—amongst whom were Lord Roberts, V.C., and the Lord Chancellor of Ireland, Lord Langford, Lord Justice Fitzgibbon, &c.

Mr. Dallas Pratt, hon. secretary of the Dublin Centre of the St. John's Ambulance Association, gave an account of that body and its work. The Order of St. John, and its offshoot, the St. John Association, dates from about the year 1014, when some Italian pilgrims, horrified at the cruelties practised on children by the Saracens, founded an association for their protection, and for the care and treatment of the sick. In the year 1095 the Pope issued a Bull making the organization an order of military monks, who were known as the Order of St. John of Jerusalem, and who won for themselves great renown during the Crusades. When the Christian Powers were compelled to withdraw from the Holy Land, the Order established its headquarters at Cyprus, and later at the Island of Rhodes, which they fortified, and where they established a hospital. After sustaining two sieges—one in the year 1480, by Solyman the Magnificent, and the other in 1522—the Order removed to Malta, where they remained until 1802, when they were dispossessed by Napoleon. Buonaparte sacked the island, taking, amongst other things, a relic said to be the hand with which St. John the Baptist had baptised the Saviour. The members of the Association then took refuge in England, and in the year 1874 started the St. John Ambulance Association.

Lord Justice Fitzgibbon told the story of the nurses' adventure in Inniskea, with which our readers are acquainted, and then Mr. Davies, Senior Knight of Justice of the Order, representing His Royal Highness the Prince of Wales, handed each of the nurses her diploma, after having said, "It is now my duty to receive into the Order those ladies to whom the rank of Honorary Serving Sisters has been granted, namely, Elizabeth Carson, Sarah Jane Caldwell, Elizabeth Doyle, Flora Kathleen Fitznaurice, Honora Kenny, Kathleen Mary Kinsella, Frances Dorothy Macalister, Margaret M'Munn, Mary Simpson, Grace Simpson, May Talbot. By the authority of His Royal Highness the Grand Prior of the Order of the Hospital of St. John of Jerusalem in England, I receive you as Honorary Serving Sisters of that Order. Her Excellency, as representing the Queen, will now invest you with the decoration pertaining to your rank in the Order which her Majesty has been graciously pleased to permit you to accept and wear."

Each nurse was then presented to the Lord Lieutenant and Countess Cadogan, who shook hands with them warmly; and the Countess then attached the badge on the left shoulder of each recipient.

After this Mrs. Tracy, the lady manager of the City of Dublin Nursing Institution—to which the sisters belong—was presented to the Viceroy and Countess Cadogan.

### "THE HOSPITAL" CONVALESCENT FUND.

We have great pleasure in acknowledging a subscription of £2 to the above fund from "Anon."

### Appointments.

### MATRONS.

Bristol (Royal) Hospital for Sick Women and Children.—Miss Beatrice Lina Colborne was appointed Matron of this hospital on August 26th, 1898. She was trained at the Royal Children's Hospital, Glasgow, and the Radcliffe Infirmary, Oxford. She has successively held the following appointments: Sister of the men's ward, Radcliffe Infirmary; sister of the children's block of the same institution; superintendent of the nursing home, St. Mary's, Paddington; and matron of the Children's Hospital, Queen Street, Belfast.

Chichester Infirmary.—Miss Annie Speck, who was trained at the Nightingale School, has been appointed Matron and Superintendent of Nurses here. She has been on the staff of St. Thomas's Hospital, and sister at the Sussex County Hospital, Brighton.

Wakefield Union Infirmary.—Miss Agnes J. Last was on August 24th, 1898, appointed Superintendent Nurse of this institution. She is at present superintendent of the Union Infirmary, Stapleton, Bristol.

### Minor Appointments.

Boston Hospital, Lincolnshire.—On August 20th, 1898, Miss Edith Northam was appointed Charge Nurse of this hospital. She was trained at the General Hospital, Tunbridge Wells, and has been staff nurse at the Royal Hospital, Richmond. She has worked with the Bedfordshire Hospital Trained Nurses' Institute, and at Maidstone during the typhoid epidemic.

Royal Albert Edward Infirmary, Wigan.—On August 28th, 1898, Miss Jean McKerrow, who was trained at Charing Cross Hospital, was elected Night Sister of the above infirmary. She has previously held an appointment as staff nurse and taken holiday sister's duties at her training hospital.

Chichester Infirmary.—Miss Florence Parkes, who resigned in November, 1897, has been reappointed Sister of the theatre and men's wards of this infirmary. She was trained at the Sheffield Royal Hospital.

Seamen's Hospital, Greenwich.—Miss Lucy Mary Paine has been appointed Surgical Sister here. She was trained at Queen's Hospital, Birmingham, and has since been night superintendent of the Walsall and District Hospital.

### Presentations.

Miss Compton, for seven and a half years nurse of the Newport District Nursing Society in the Isle of Wight, has lately resigned her work. She has been presented with a purse containing £10 and an illuminated address bearing the borough arms and the following inscription: "Presented to Nurse Compton, with a purse of money, by the undersigned subscribers and friends, on the occasion of her leaving the Isle of Wight, as a token of their appreciation of her good and faithful service for more than seven years." There are more than sixty names subscribed, headed by those of the Mayor and Mayoress.

A silver tea service and tray have been presented to Nurse Jessie Henry by her sister nurses as a token of esteem and farewell on the occasion of her leaving the Royal Scottish Nursing Institution, 69, Queen Street, Edinburgh, to take up her duties as matron of Peebles Fever Hospital.

Miss I. M. Eastmond, who has resigned the matronship of Brentwood Cottage Hospital, was presented on leaving with a testimonial and a purse of twenty-five sovereigns by friends and patients, past and present.

### Wants and Workers.

Will any reader of The Hospital care to exchange "Hospital Sisters and Their Duties," by Miss Lückes, and "The Midwives' Pocket-Book," by Honnor Morten, for "Helps in Sickness and in Health: Where to Go and What to Do," by Sir H. Burdett—new edition?—*Nurse Constance*, Farnborough, Banbury.

### A Book and its Story.

"MEDICAL WOMEN IN FICTION."

A WRITER in a recent number of *Blackwood's Magazine* gives us an interesting article on the above subject, which, coming as it does at a moment when public attention has been directed to an epoch-making event in the history of the London School of Medicine for Women, has a particular interest for us all. Henceforth, as the writer comments, "even to the most reactionary mind, the medical woman is neither a fad nor a fancy, but an established fact. The position of the medical woman is assured to-day, and one is apt to forget the great fight of afflictions through which she fought her way to attain this end." Before commencing our brief review of medical women as they are shown in fiction, it would be as well to glance at the history itself of the band of women who, in the earlier part of the present century, waged a war of independence in regard to the medical degree. Miss Garrett's is perhaps the best-known name among this group of strugglers, the young woman who had the presumption to pass, in spite of all the difficulties thrown in her way, the only English medical examinations then open to her sex, *i.e.*, those of the Apothecaries' Hall; it is still in the memory of many of us how the door through which she passed was promptly shut in the faces of any who might wish to follow her example by those who stood aghast at her success. Next, we remember how Miss Jex Blake sought in vain to obtain admission to the London University examinations, and on turning her attention to those of Edinburgh, was informed by one of the Professors to whom she appealed that "no decent woman would apply to him to study medicine." Undeterred even by this *obiter dictum*, a little band of ladies gained leave to enter for the preliminary examination, after passing which they were allowed to matriculate, but failing to take warning by the fate of Miss Garrett, they passed so well that a deliberate attempt was made to revoke the promise given. The effort failed, and for several months the lady medical students worked under the happiest auspices, until the result of the class examinations awoke the slumbering animosities once more. The women distinguished themselves, too, signally, and it was thought fit to punish them by refusing Miss Pechey the scholarship in chemistry which she had earned, and by denying her also admission to the university laboratory. "The refusal of the scholarship," in the words of the article now under review, "had its effect for good on those who followed in Miss Pechey's footsteps, one result of the preferential dealing being an outburst of public scorn—and certain physiological classes from which ladies had previously been excluded, were opened to them—but the professors still continued their stern refusal to allow their attendance at the anatomy lectures, thus necessitating the study of anatomy and clinical surgery at Stationers' Hall. Closely on this followed the objections raised by doctors to ladies practising in the Town Infirmary at Edinburgh. Litigation was resorted to; time and money wasted." Finally, Edinburgh was given up as an attempted ground for the career of lady medical students, and continental universities, with less narrow prejudices, opened their doors to the women who offered themselves for the prescribed medical curriculum.

Thus, though in a much abbreviated form, does the writer of the article recall to us the history of the many struggles of medical women in the past, and thus, in stirring words, are told the personal struggles of Rhoda Gale in Charles Reade's "Woman Hater,"* in the story she recounts to Harrington Vizard in Leicester Square. "Dr. Rhoda shared one characteristic with the majority of medical women in fiction"—she is not at all meek; indeed, she is an essentially combative temperament. We must own that we have not observed this peculiarity to be general among lady doctors in real life; but then we have never attempted to convince them that it was either impossible or improper for them to have studied medicine. . . . "Rhoda Gale is happy in the listener to whom she recounts her woes, and she finds in the Woman Hater the friend she needs." We leave her established as the unofficial health officer of the district, secure, in spite of her unregistered condition, under the autocratic sway of Vizard, who succeeds even in obtaining for her permission to visit the local infirmary.

Different indeed is the heroine of "Sweethearts and Friends,"* who is one of those beauties almost peculiar to fiction who are first seen as "shy awkward girls, with red hands and untidy hair . . . and who, in the course of a few years, develop into beings of surpassing loveliness." Dr. Amy, whose career is here related by Maxwell Gray, commences her work at anatomy and physiology at an early date. "To aid her studies Amy kept a human skull under her pillow, which appears to the ordinary mind about the most unsafe place she could have chosen in the daytime and the most uncomfortable at night." Amy has her difficulties, but they are more nearly connected with the views taken by certain members of her family than with the difficulties met with by Rhoda Gale in the course of her professional training. Amy's lot fell in pleasanter places, and she was able "to take advantage of the newly-founded School of Medicine for Women, the modest commencement of the great enterprise of to-day." At the end of the book Dr. Amy marries a very remarkable man, whom one eventful day, with a wild cry of "With him or for him," she had rescued from a burning house, "swarming up a rope, and reaching the tottering balcony in a few seconds." Amy's husband, we are told, never forgot the cry as narrated above. But despite much that is so melodramatic and improbable in "Sweethearts and Friends," the writer according to our article under review, remarks pertinently that Maxwell Gray shows in her book "an agreeable rallying turn."

Far more seriously does the author of "Doctor Victoria"† take his heroine, the one specialist we have met with among the medical women of fiction, who directs her attention to diseases of the eye. "Her male confrères have no objection to meet her in consultation, and the hostility of outsiders is disarmed by her attachment to music and needlework and the care she bestows on her looks and her dress." With all respect for Major-General Alexander's literary product, it strikes one as being devoid of all sense of humour.

In Mr. George Knight's "Winds of March"‡ we are presented with a somewhat extraordinary type of American lady doctor, who has many attainments other than her professional career. "Bab is an artist of extraordinary power, a marvellous musician, a captivating singer, and quite casually and by the way an M.D. of New York."

Amongst other works of fiction which concern themselves with lady doctor heroines are "Mona Maclean, Medical Student," by Graham Travers, and "Peace With Honour," by Sidney Grear, and in "Dr. Janet of Harley Street," § by Arabella Kenealy, we have yet another instance. Dr. Janet is "senior physician of the Minerva Hospital for Women, dean of the medical school attached to it, and lecturer at various colleges, and besides all this she has a large practice which ranges from Royalty to the poorest of the poor. Her chief characteristic physically is, we regret to say, a certain stalwart shapelessness, and morally, a habit of telling unpleasant truths in a deep voice."

Such, in merest outline, are the principal studies of

* "The Woman Hater." By Charles Reade.

* "Sweethearts and Friends." By Maxwell Gray.
† "Doctor Victoria." By Major-General Alexander, C.B.
‡ "Winds of March." By George Knight.
§ "Dr. Janet of Harley Street," By Arabella Kenealy.

medical women in fiction, as discussed in the interesting article in the current number of our contemporary, and though perhaps in no one instance can one be especially interested in these women as heroines, yet they serve the useful purpose of forming an unconscious history of the troublous times which preceded the times of peace in which the lady medical student of to-day happily finds herself. "Mona Maclean,"* whose character is described by the pen of Graham Travers, marks, as clearly as does the Royal recognition of which we have already spoken, the close of the transition period. The medical woman has vindicated in the eyes of others her right to live, she has now to justify her existence in her own.

* "Mona Maclean, Medical Student," By Graham Travers.

### Everybody's Opinion.

[Correspondence on all subjects is invited, but we cannot in any way be responsible for the opinions expressed by our correspondents. No communication can be entertained if the name and address of the correspondent is not given, as a guarantee of good faith but not necessarily for publication, or unless one side of the paper only is written on.]

### WHAT ONE NURSE WOULD HAVE LIKED.

IN regard to workhouse infirmaries "E. F." writes that the following things are wanted: A sufficient supply of sheets and body-linen always at hand and under her own care; also a night nurse to give the sick nourishment and other attentions needed during the long and weary hours of the night. Some people think that the aged and helpless in sickness do not need night nursing. How strange it would be if they did not. Next, how nice it would be if, when a change of food was necessary for a bad case, those responsible for the cooking did not make such a trouble of it. Of course it causes "ructions" to stir up places that have been long dormant, and anyone who dares to attempt it may look for unpleasantness.

### LADIES *V.* GENTLEWOMEN.

"A DOCTOR'S DAUGHTER" writes: Will you allow me to add a few remarks with regard to the subject "Ladies *v.* Gentlewomen"? I do not at all agree with "Nurse" in her opinion that the proper definition of gentlewomen is "women of refined and gentle natures, whose minds are pure and holy." It is ridiculous on the face of it, and no one by any stretch of imagination would call a working man who has a refined and gentle nature a gentleman, or a refined and gentle housemaid (such as I have often come across in my varied experience in nursing) a gentlewoman. Truly "Nurse" is quite out of it, and if she will kindly turn to her dictionary she will there find the true and correct definition of gentlewomen. I, for one, am thankful to think that we have matrons who will only take gentlewomen on to their hospital staffs, and shall hail the day when the last "servant-class" nurse (however refined and gentle) is refused admission into our splendid hospital wards. This must be done if the reputation of the British nurse is to be maintained.

### Death in Our Ranks.

THE Birmingham and Midland Counties Training Institution for Nurses has recently sustained a severe loss in the death of the Lady Superintendent, Mrs. Diamond. She was the widow of Francis H. Diamond, who died at Orleans, in France, in 1866. After the death of her husband, Mrs. Diamond entered the Evelina Hospital and trained as a nurse; she then became Superintendent of the Nurses' Institute, Glasgow, and on May 28th, 1875, was selected for the post which she retained until her death, a period of 23 years. The chairman and committee have passed a resolution of regret and appreciation of herself and her services, and numerous tokens of affection and esteem have been received by her friends.

### Sister Ellen of the Walsall Hospital.

BY ONE WHO KNEW AND LOVES HER.

"THE old order changeth, giving place to new." Such was my thought on revisiting Walsall Hospital, with its enlarged and renovated wards, capable matron, house surgeon, large staff of visiting doctors, and numerous nurses—all so up-to-date. But as one who remembers the "old order" I am glad to give some record of the work of Sister Dora's immediate successor—Sister Ellen Hall. It may not be known to many that Sister Dora never worked in the new building; and that added to the agony of the disease which ended her life was the anxiety about who should succeed her in the care of the Walsall folk whom she loved so well; her anxiety was laid to rest by the offer of Sister Ellen to continue the work. The only nursing Sister Dora herself received was during the last fortnight of her life, when Sister Ellen did all that was possible to give her some relief. "Oh," she said, "if my poor patients can be nursed like this, I am content!"—and she was fully justified in her trust. Sister Ellen had had two years' hospital training, one at King's College Hospital under the old sisterhood of St. John's House, and one year as sister of two important wards in the "London." Added to this Dr. Maclachlan gave her valuable surgical instruction, with the result that for the eleven years she worked at Walsall there was no house surgeon, all simple fractures (amounting in that time to nearly 1,000), burns, &c., were attended to by her. None who worked with her could fail to admire her self-denying life and that of her assistant "sister"—always at the service of the poor injured patients who were brought in at any hour of the day or night. It is said the diet was not generous in those old days—that may be, but economy was a necessity in the hospital, and no luxury was enjoyed by the sisters—the dietary was the same for patients, lady pupils, and sisters. Though in weak health Sister Ellen gave her services gratuitously for eleven years, and when she felt her power of active service was gone she resigned, dying after two years of failing health. She now rests from her labours in the cemetery at Ventnor. In 1885 she founded a seaside convalescent home in North Wales, primarily for patients from the Walsall Cottage Hospital, but open to others if there were vacancies. This is still carried on, but with a certain amount of anxiety about the annual income. Would that some grateful Walsallite would endow it, or enable a house to be purchased in memory of the good work she did at Walsall as Sister Dora's successor.

### The Milkmaid Nurse.

SOME of the comments of the *Coleraine Herald* on our article of the above title are really too amusing to pass unnoticed. Our readers are certain to enjoy the joke. The *Coleraine Herald* disapproves of our remarks, but after all only repeats our complaint as follows:—

"To show our disapproval of THE HOSPITAL man's sarcasms we have taken the extreme step of handing them over to our poet to work his will on them. He has boiled them down into some very bad verses, of which we give a couple.

THE SONG OF THE WOULD-BE NURSE.

I haven't one credential, but that is not essential;
 I never thought of nursing until now;
For working with the sick I am not the one to pick—
 But few know better how to milk a cow.

My footfall isn't light; my face is seldom bright;
 My voice is somewhat strident, I allow;
And I doubt if I'd be able to read a doctor's label—
 But what of that when I can milk a cow?"

### For Reading to the Sick.

**Verses.**

"LIVING WATERS."

THERE'S no one to whom's not given
Some little lineament of heaven,
Some partial symbol, at the least, in sign,
Of what should be, if it is not, within,
Reminding of the death of sin
And life of the Divine.

Glory to God ! that I am born
Into a world where palace gates
So many royal ones adorn—
Heaven's possible novitiates !

Princely ye are, each one, to me,
Each of secret, Kingly blood,
Though not inheritors as yet
Of all your own right royal things.
Yet are ye angels in disguise,
Angels who have not found your wings.

—*Sutton.*

Ye have not sown in vain !
Though the heavens seem as brass,
And piercing the crust of the burning plain
Ye scan not a blade of grass.

Yet there is life within,
And waters of life on high ;
One morn ye shall wake, and the spring's soft green
O'er the moistened fields shall lie.

—*Author of " The Three Wakings."*

Oh, Christ He is the fountain,
The deep sweet well of love !
The streams on Earth I've tasted,
More deep I'll drink above ;
There, to an ocean fulness,
His mercy doth expand,
And glory, glory dwelleth
In Immanuel's land.

—*Cousins.*

**Reading.**

The words "living water" occur in two remarkable passages of St. John's Gospel, passages widely separated in time and circumstance, but closely united in spiritual significance by this phrase, and that, too, in a way which makes the second passage the true sequel and development of the first. The Lord Jesus tells the woman of Sychar that had she known the gift of God, and known Him who spoke to her, she would have asked, and He would have given living water; and that this water would have precluded all thirst for ever; and that it would prove to be, within its recipient, a fountain of water, of water not stagnant, but "springing, leaping unto eternal life . . ." He invites all who thirst to "come to Him and drink." And by this He means "to believe on Him." And He assures His hearers that as this is done the result shall be not merely a reception of living water, but such a reception as shall be an overflow. Out of the drinker, out of the believer, " shall flow rivers of living water. And this spake He of the Spirit, which they that believed on Him should receive." . . . The man who really drinks of Christ, drinking of the Spirit, shall assuredly be a conveyer, a conductor for the Spirit, for Christ, to others. . . . The really and livingly spiritual man shall be a spiritual blessing, a spiritual power. The thought requires, of course, a reverent caution. . . . Our work in the matter is to drink, is to believe ; to live by faith on the Son of God, who obeyed and died for us and liveth in us. His undertaking is that we shall be in proportion, aqueducts for His living water.—*H. E. C. Moule.*

### Notes and Queries.

**The contents of the Editor's Letter-box have now reached such unwieldy proportions that it has become necessary to establish a hard and fast rule regarding Answers to Correspondents. In future, all questions requiring replies will continue to be answered in this column without any fee. If an answer is required by letter, a fee of half-a-crown must be enclosed with the note containing the enquiry. We are always pleased to help our numerous correspondents to the fullest extent, and we can trust them to sympathise in the overwhelming amount of writing which makes the new rules a necessity.**

**Every communication must be accompanied by the writer's name and address, otherwise it will receive no attention.**

*Co-operation's Commission.*

(211) I belong to a large and successful nurses' co-operation, working from a private nursing home. I have to pay commission on all my cases, on those I obtain myself as well as those found for me by the co-operation. Is this the usual thing?—*Nurse Agnes.*

Yes; it is usually so. The reason is plain. The institution would have provided a case if the nurse had waited, and commission is needed to meet expenses.

*Male Nurses.*

(212) (1) Can you tell me of any place in England where male nurses are trained ; (2) and, when qualified, is there any difficulty of obtaining a post?—*Ambulance.*

The National Hospital for the Paralysed and Epileptic, Queen's Square. Bloomsbury, is the only hospital in England where men receive instruction in sick nursing. Many of the asylums, however, give sick training in addition to the instruction in mental work. (2) There is a good demand for male nurses.

*Vermin.*

(213) What disinfectant can I carry in my box as a preventative against bugs ? I am engaged in private work, and have a great horror of them. —*Private Nurse.*

Use carbolic soap for toilet purposes and in your box Keating's insect powder, and brush your clothes frequently.

*Vacant Accommodation.*

(214) Which is the right way to act when a patient asks if you will have a bed-room vacant in a week or two's time in a nursing home? —*C. J.*

In the event of a patient requiring you to keep a room empty for his or her convenience you should ask a retaining fee which will cover the cost of the room to you for the period of waiting.

*Books for a Probationer.*

(215) Can you name the most suitable books on medicine and surgery for a probationer to read?—*Pater.*

The Scientific Press, London, publishes a series of books especially adapted to a nurse's requirements. " Nursing : Its Theory and Practice," by Lewis (price 3s. 6d.); " Elementary Physiology for Nurses," by Marshall (2s.); and " Elementary Anatomy and Surgery for Nurses," by McAdam Eccles (2s. 6d.), are all useful and suitable.

*Hip Disease.*

(216) Would you kindly let me know if there is any home where a boy of nine years, with hip disease, could be received ? The father could pay a small sum weekly. The child is in a leather splint, and uses crutches.—*M. (Shepherd's Bush).*

See list of charities for chronic and incurable cases in " Burdett's Hospitals and Charities," 5s, from the Scientific Press, London. But does not the child require treatment?

*Immediate Work.*

(217) Kindly give me the names of any hospital in Liverpool where they are likely to take a probationer immediately. Salary needed at once.—*A B C.*

See our advertisement columns. They are generally acknowledged to be the best public register of vacant appointments for nurses. Consult also " Burdett's Official Nursing Directory " (5s., from the Scientific Press, London), and select the training schools in Liverpool that seem most likely to suit you, and then apply direct to the Matron.

*Dispenser's Certificate.*

(218) I have been assisting in the dispensing of this hospital for some time, under the doctor's supervision. I should like to obtain a diploma, and do not know how to set about it.—*Matron.*

You should take the " certificate of qualification to act as an assistant in compounding and dispensing medicines" of the Apothecaries' Society, Blackfriars, London, E.C. Write to the Secretary for particulars of examination, the fee for which is £2 2s.

*Recovery of Fees.*

(219) Having seen in your issue of August 19th about nurses' fees will you kindly answer me the following questions: I was engaged to nurse an invalid case at £70 per year, and if I stayed six months a bonus of £10 down would be given as well. I have now completed my five months, but the patient is slowly dying, and from appearances cannot last above a week or two. If he should pass away before my six months are up, am I entitled to the £10, or can I claim a month's notice or wages, having entered well into the last month?—*Companion.*

This is a question for a solicitor. The arrangement seems to have been so far special and out of the common run that no question of professional or trade custom is likely to arise. In default of a written agreement, we should doubt very much whether the bonus could be recovered. There can, however, be hardly any doubt that a certain notice, or wages in lieu of notice, must be given, the length of the notice depending much upon the frequency with which the wages were paid, weekly, monthly, or quarterly.

*The Use of the Catheter.*

(220) Can you tell me of any work published on the use of the catheter? —*E. H.*

No special work on this subject is published for the use of nurses. A single practical demonstration would be more valuable than many books. This applies even more to the use of the male than of the female catheter.

